# “LET’S TALK TECH” IN THE ALGORITHMIC AGE: ENGAGING PEOPLE LIVING WITH DEMENTIA IN TECHNOLOGY USE DECISIONS

**DOI:** 10.1093/geroni/igae098.1440

**Published:** 2024-12-31

**Authors:** Clara Berridge, Natalie Turner

**Affiliations:** University of Washington, Seattle, Washington, United States; University of Washington, Seattle, Washington, United States

## Abstract

The availability of technologies to support dementia care is outpacing our understanding of how to help families navigate use decisions and align with the values and preferences of the person living with dementia. This is an ethical problem made urgent by efforts to incorporate data from the home into AI healthcare applications where transparent disclosures are still aspirational. The misperception that people living with mild-to-moderate dementia cannot or do not want to be involved in decision making about their care is widespread, yet failure to enable decision making participation where possible reflects ageism and ableism. An added barrier to involvement in decision making about digital technologies is communication about the technologies themselves. Findings will be presented from a mixed-methods study of the first of its kind tool to facilitate empowered, shared decision making by people living with dementia and care partners about technologies that use a range of data (e.g., audio, visual, location) and include companion robots. Together with primary care partners, twenty-nine participants living with mild Alzheimer’s disease (AD) learned about, discussed, and documented their preferences for each of four categories of technologies through the Let’s Talk Tech intervention. Individual preferences were not consistent across technologies and varied considerably, supporting the need for personalized decision making. Most participants living with mild AD reported that they wanted (vs. unsure or do not want) each of five control-enhancing options, including being informed if a technology is used to monitor them and to have the ability to pause monitoring for privacy.

